# Nanomorphological and mechanical reconstruction of mesenchymal stem cells during early apoptosis detected by atomic force microscopy

**DOI:** 10.1242/bio.048108

**Published:** 2020-03-30

**Authors:** Xuelian Su, Haijing Zhou, Guangjie Bao, Jizeng Wang, Lin Liu, Qian Zheng, Manli Guo, Jinting Zhang

**Affiliations:** 1 Key Laboratory of Stomatology of State Ethnic Affairs Commission, Northwest Minzu University, Lanzhou 730030, China; 2 Key Laboratory of Mechanics on Disaster and Environment in Western China, The Ministry of Education of China, Lanzhou University, Lanzhou 730000, China; 3 Key Laboratory of Oral Diseases of Gansu, Northwest Minzu University, Lanzhou 730030, China

**Keywords:** Biomechanics, Nanomorphology, Apoptosis, Bone marrow mesenchymal stem cells, Atomic force microscopy, Macromolecular crowding

## Abstract

Stem cell apoptosis exists widely in embryonic development, tissue regeneration, repair, aging and pathophysiology of disease. The molecular mechanism of stem cell apoptosis has been extensively investigated. However, alterations in biomechanics and nanomorphology have rarely been studied. Therefore, an apoptosis model was established for bone marrow mesenchymal stem cells (BMSCs) and the reconstruction of the mechanical properties and nanomorphology of the cells were investigated in detail. Atomic force microscopy (AFM), scanning electron microscopy (SEM), laser scanning confocal microscopy (LSCM), flow cytometry and Cell Counting Kit-8 analysis were applied to assess the cellular elasticity modulus, geometry, nanomorphology, cell surface ultrastructure, biological viability and early apoptotic signals (phosphatidylserine, PS). The results indicated that the cellular elastic modulus and volume significantly decreased, whereas the cell surface roughness obviously increased during the first 3 h of cytochalasin B (CB) treatment. Moreover, these alterations preceded the exposure of biological apoptotic signal PS. These findings suggested that cellular mechanical damage is connected with the apoptosis of BMSCs, and the alterations in mechanics and nanomorphology may be a sensitive index to detect alterations in cell viability during apoptosis. The results contribute to further understanding of apoptosis from the perspective of cell mechanics.

## Abstract (precis)

**Summary:** Cellular mechanical damage is connected with the apoptosis of BMSCs, and the alterations in mechanics and nanomorphology could be used as a sensitive index to detect alterations in cell viability during apoptosis.

## INTRODUCTION

Stem cells exist extensively in various tissues, such as bone marrow, fat, muscle, hair follicle, dental pulp and nerves, of organisms. When these tissues are damaged, the body can repair and heal itself. Improved understanding of stem cells has revealed that these cells play an important role in tissue repair and regeneration, multidirectional differentiation, embryonic development and pathophysiology of disease (Wu et al., 2007; Sasaki et al., 2008; Amoh et al., 2009). When large numbers of stem cells are damaged, the body's ability to repair itself is weakened. For example, chemotherapy agents target rapidly proliferating cancer cells while also inhibiting fast-growing stem cells in patients. Those suppressed cells fail to repair the damaged tissue in time, leading to side effects from chemotherapy, such as myelosuppression, immunosuppression, inflammation and alopecia (Ewald et al., 2010; Demaria et al., 2017). In addition, increasing evidence has demonstrated that bone marrow mesenchymal stem cell (BMSC) transplantation and tissue engineering are promising therapeutic strategies for acute brain damage (Tang et al., 2015), spinal cord injury (Rosner et al., 2012), heart injury (Gao et al., 2017) and bone and cartilage defects (Liu et al., 2010; Dahlin et al., 2014). BMSCs were recruited to the ischemic heart and repaired injured myocardium after heart infarction (Abbott et al., 2004). Newly generated cells derived from BMSCs could replace dead cells in injured hearts and vessels. However, BMSC transplantation and engineered tissue construction are hampered by poor survival of stem cells in the recipient sites (Wang et al., 2013; Li et al., 2017). Apoptosis has become a bottleneck for the further development of stem cell transplantation and tissue engineering. Therefore, understanding cell events during apoptosis of stem cells is imperative.

The apoptosis of stem cells plays an important role in various physiological activities, including embryonic development, homeostasis in normal tissues and aging (Brill et al., 1999; Tower, 2015). However, abnormal apoptosis of stem cells can lead to disease and/or poor self-repairing capability. One study found that radiation induced apoptosis of BMSCs through mir-22-exotrophic stress, which affected the repair and regeneration of bone tissue in irradiated sites (Liu et al., 2018). Moreover, the major metabolite 2,5-hexanedione of the organic solvent n-hexane, which is widely used in adhesive preparation, paints (including oil-based), varnishes and the food industry, can induce apoptosis of BMSCs via the mitochondria-mediated caspase-3 pathway (Sun et al., 2018; Chen et al., 2015) and nervous lesions (Cui et al., 2007). Some chemotherapy drugs, such as arsenic trioxide, which significantly inhibit acute leukemia cells, induce mesenchymal stem cell (MSC) apoptosis through the mitochondria-mediated caspase-3 pathway in the patient's body (Cai et al., 2010). BMSCs were also significantly damaged by chemotherapy agents in colorectal cancer (Cao et al., 2008). Therefore, an in-depth understanding of stem cell apoptosis is of great significance for recognizing disease occurrence, researching drug toxicity and the cell's ability to repair itself, stem cell transplantation and tissue engineering. In recent years, the molecular mechanism of apoptosis in BMSCs (Chen et al., 2015; Cui et al., 2007; Cai et al., 2010) and malignant cells (Li and Yuan, 2008; Jeong et al., 2014) has been extensively studied. However, the cellular mechanics and nanomorphology during early apoptosis of stem cells have rarely been studied. Therefore, there is a need to understand the mechanical events of stem cell apoptosis at the nanoscale.

Bio-type atomic force microscopy (AFM) is an effective technique to directly measure the mechanical properties of biological specimens and to quantify the cell morphological changes at the nanoscale level under physiological conditions in a real-time, label-free manner (Müller and Dufrêne, 2008; Stark, 2007; Stewart et al., 2012). The mechanical properties of cancer cells have been extensively explored by AFM during apoptosis (Zhang et al., 2014; Bai et al., 2017). Our previous studies about apoptosis recognized that it is not only closely connected with biological signal cascades but is also related to cellular mechanical remodeling (Zhang et al., 2016; Su et al., 2019). However, alterations in mechanics and nanomorphology during apoptosis have rarely been studied.

Therefore, the present study selected BMSCs to establish an apoptosis model to investigate the alterations in mechanical properties and nanomorphology during early apoptosis. It was found that the elastic modulus and volume of the cells significantly decreased whereas the surface roughness obviously increased during the first 3 h of cytochalasin B (CB) treatment. These alterations preceded the exposure of biological apoptotic signal, phosphatidylserine (PS). Further observation showed that the F-actin fragments filled the cell, and some of them passed through the membrane and stayed on the cell surface with disruption of the cytoskeleton. These findings suggested that cellular mechanical damage is connected with the apoptosis of BMSCs.

## RESULTS

### Identification of BMSCs

**Fig. 1.**
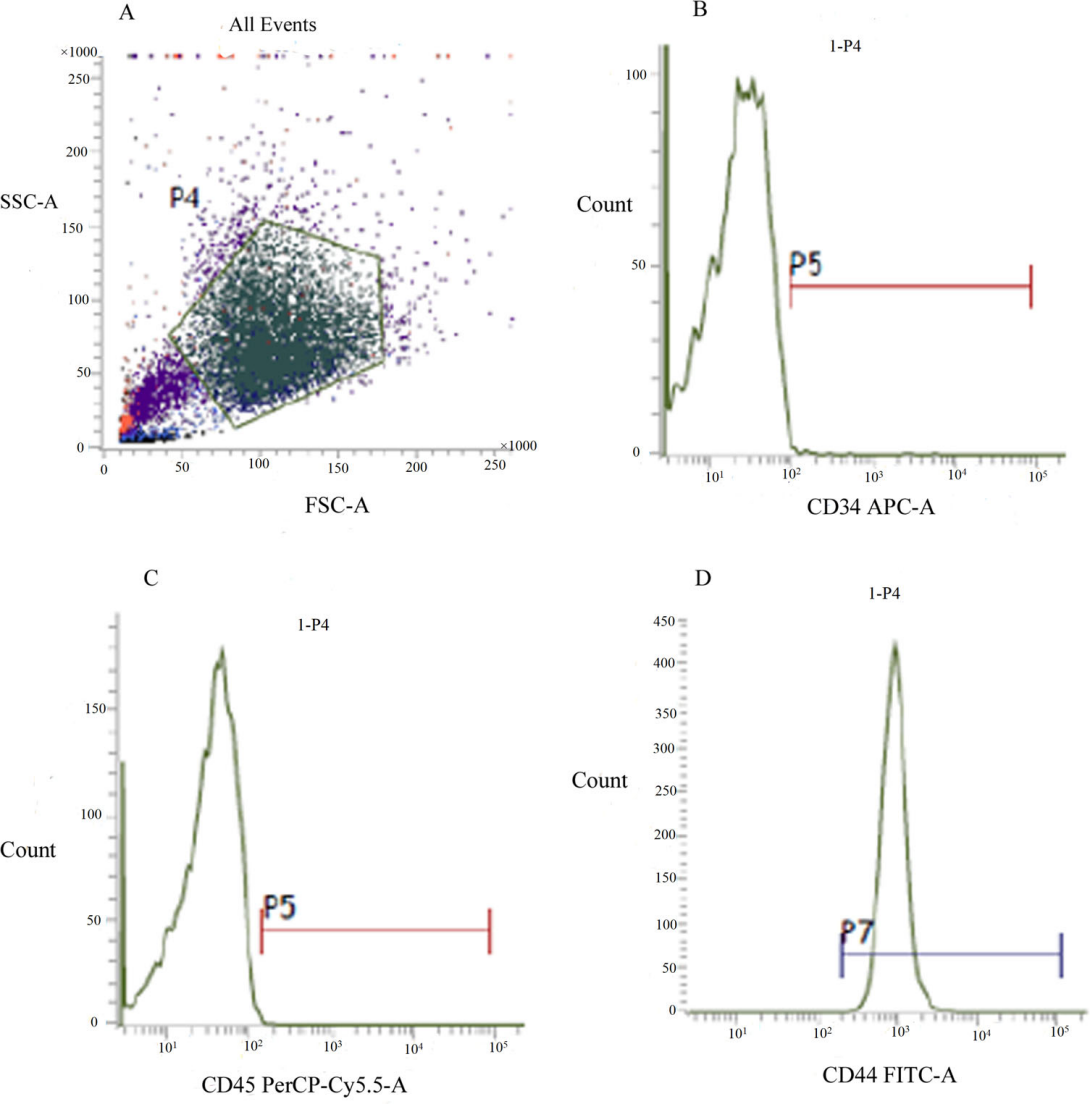
**The flow cytometry (FCM) analysis of BMSCs molecular surface.** The pentagonal frame represents the cell area selected for FCM analysis (A). The expression of hematopoietic stem cell markers CD34 and CD45 were negative (B,C). Whereas, the expression of stem cell marker CD44 was strongly positive (D).

According to the control groups, the appropriate cell area was selected for flow cytometry (FCM) analysis (Fig. 1A). The results showed that the expression was negative for the hematogenous markers CD34 and CD45 (0.43% and 0.74%, respectively) on the surface (Fig. 1B,C) and strongly positive for the stem cell marker CD44 (99.45%) (Fig. 1D). These results collectively suggested that the cells isolated and cultured were indeed BMSCs and sufficiently pure to meet the experimental requirements.

### CB-induced cell growth inhibition

**Fig. 2.**
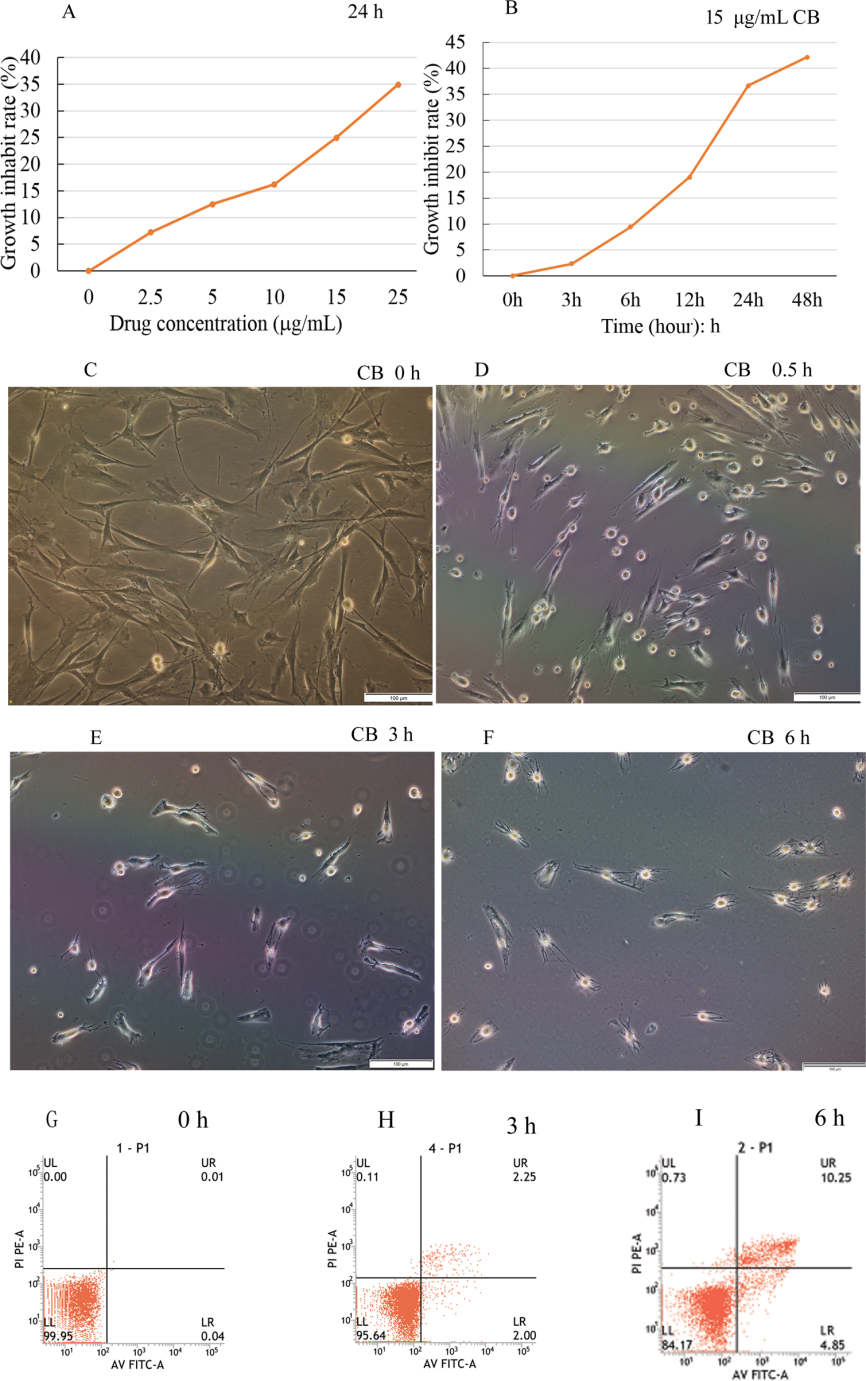
**Cell viability and apoptosis assay, morphological changes.** The cell death rate increased with the concentration and treatment time of cytochalasin B, indicating concentration and time dependence (A,B). The light micrograph revealed that the normal BMSCs shaped as long spindles or long triangles, and the boundaries were clearly visible. In contrast, the treated cells shrank little by little, became round and in some cases detached from the substrate. The cell boundaries became irregular and the adherent cells were fewer and fewer (C–F). A few apoptotic cells were detected during the first 3 h of treatment with CB (G,H), then the apoptosis rate gradually increased, reaching 15.1% at 6 h (I). Scale bars: 100 μm.

The growth of BMSCs was gradually inhibited by CB. Moreover, the inhibition showed concentration and time dependence (Fig. 2A,B). When the cells were treated with 15 μg/ml CB for 48 h, the maximal inhibitory rate was more than 40%. Therefore, this concentration was selected as the experimental concentration in subsequent procedures. The results suggested that the higher the drug concentration and the longer the treatment time, the greater the toxic effects on the cells. The light micrograph indicated the morphological alterations of cells exposed to CB. The untreated cells were well spread (shaped as long spindle or long triangle) and the cell boundaries were clearly visible (Fig. 2C). In contrast, for the treated groups, the cells shrank, rounded up little by little, and even detached from the substrate and floated in the medium. The adherent cells were fewer and fewer (Fig. 2D–F).

### Phosphatidylserine externalized on the membrane

To confirm the biological toxic effects of CB on BMSCs, apoptosis was assessed by FCM using Annexin V-FITC/Propidium Iodide (PI) double staining. A few apoptotic cells were detected in the initial 3 h compared to the control (Fig. 2G,H), and then the rate of apoptosis gradually increased to 15.1% at 6 h (Fig. 2I). The results were consistent with the cell growth inhibition rate and indicated that CB could cause the BMSC apoptosis.

### The actin cytoskeleton was disrupted by CB

**Fig. 3.**
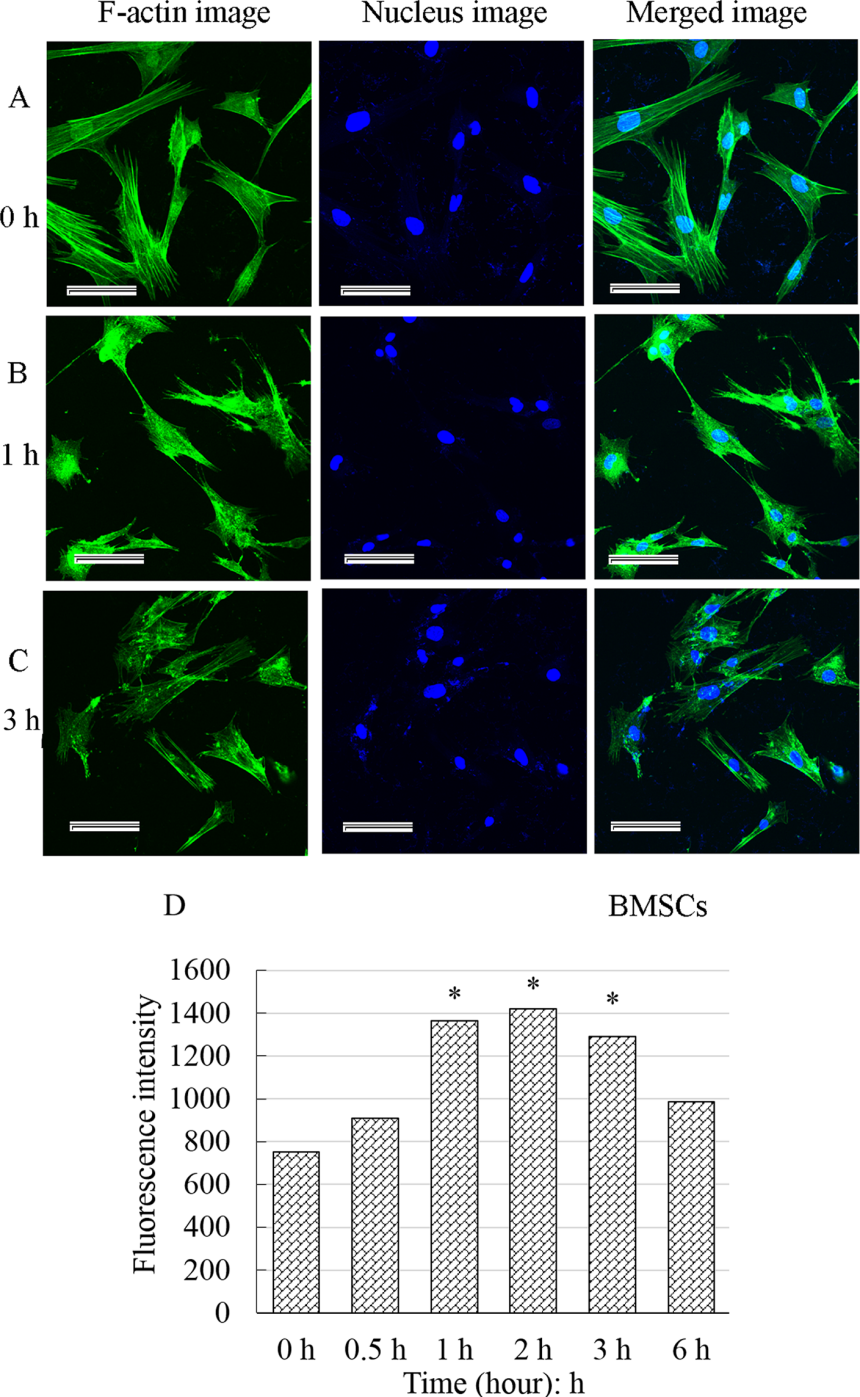
**Effect of cytochalasin B on the F-actin cytoskeleton and fluorescence intensity analysis.** F-actin uniformly distributed in the cytoplasm below the membrane. The F-actin fibers clustered into bundles (bright green fluorescence) (A). Whereas for the treated cells, the fiber bundles slowly became thinner, smaller and even disappeared and were replaced by smeared or punctate fluorescence fragments (B,C). The fluorescence intensity of F-actin significantly increased, reaching a maximum at about 2 h and then gradually decreased. The results were recorded as the mean±standard deviation and analyzed by one-way analysis of variance. *P<0.05. Scale bars: 50 μm.

The F-actin uniformly distributed in the cytoplasm below the membrane and the F-actin fibers clustered into bundles (bright green fluorescence) (Fig. 3A) in control cells. For the treated cells, the F-actin gradually depolymerized, and the green fiber bundles slowly became thinner and smaller, even disappeared and were replaced by smeared or punctate fluorescence fragments (Fig. 3B,C). The fluorescence intensity of F-actin significantly enhanced, reached a maximum about 2 h and then increasingly weakened (Fig. 3D). All these alterations revealed that the F-actin cytoskeleton was gradually interrupted by CB, and a large number of actin fragments accumulated in the cells.

### Cell surface roughness increased and were geometrically reconstructed

**Fig. 4.**
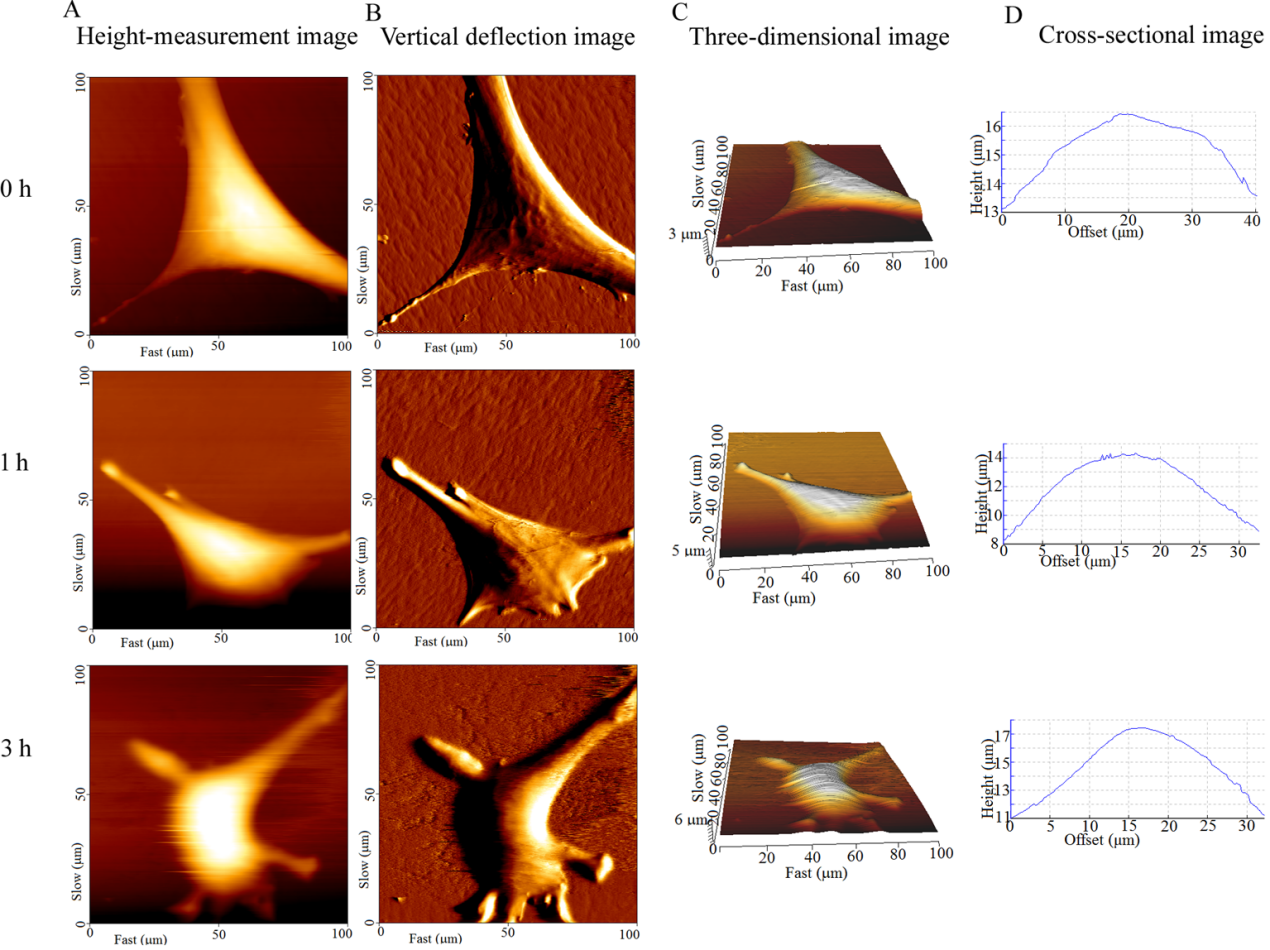
**Surface topography of BMSCs captured by AFM at different times.** Columns A–D indicated the height-measurement images, vertical deflection images, three-dimensional images and cross-sectional images, respectively. The bright area was the elevated part of the cell, where the nucleus was located (A,C). The untreated cells adhered well, and their surface was smooth. The texture of the F-actin bundles is clearly visible (B, 0 h). The surface of treated cells became increasingly rough, the periphery of the cells became irregular and the area of cell extension gradually decreased (A and B, 1 h, 3 h, respectively).

**Fig. 5.**
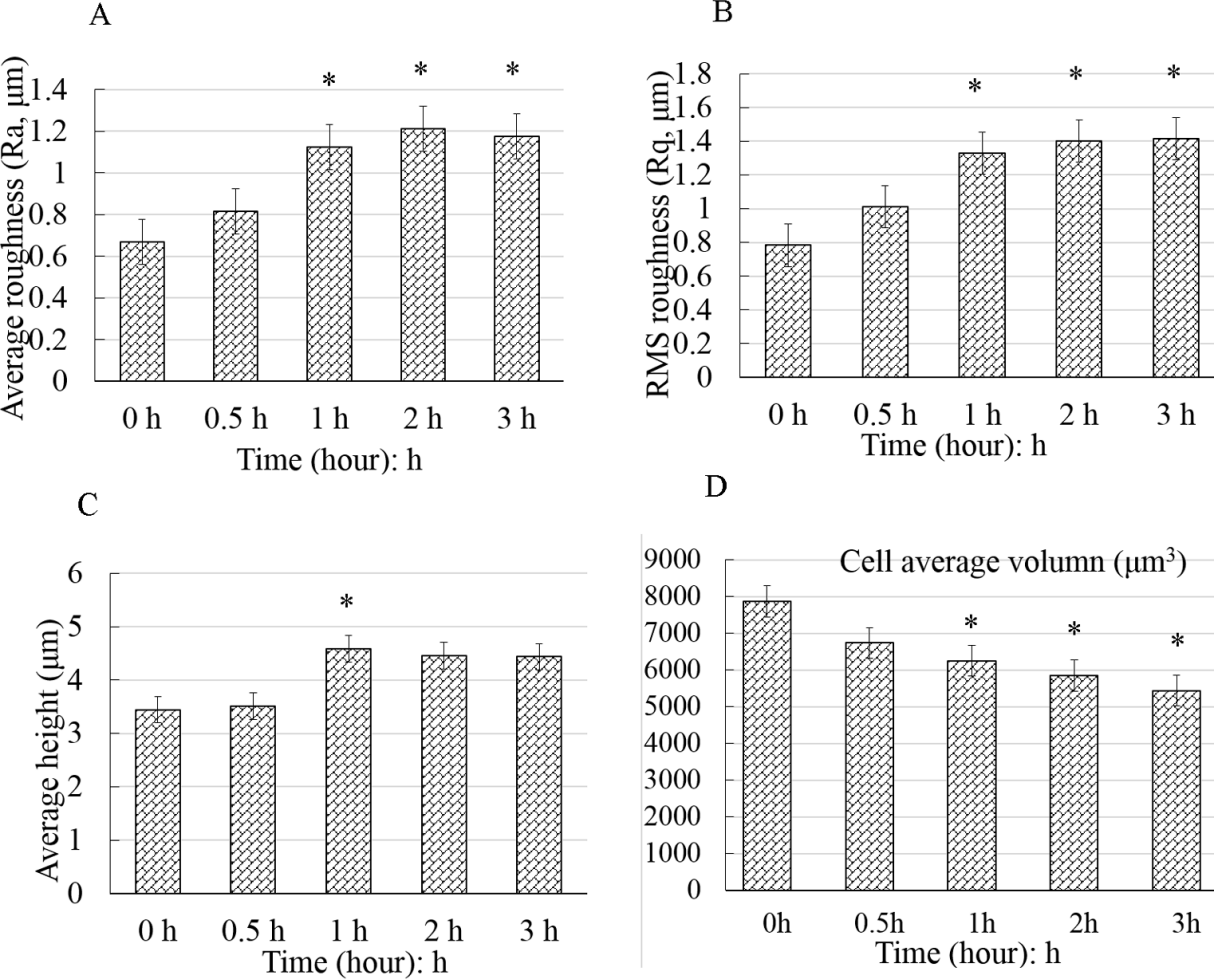
**Geometric reconstruction after the BMSCs were exposed to cytochalasin B.** The control cells displayed the least amount of roughness (A,B). The Ra and Rq values of the treated cells were significantly higher than those of the control cells (*P*<0.05). The cell height increased at first and then slightly decreased. However, the height of the treated cells was significantly higher than that of the control cells (C). The cell volume continued to decline and significantly decreased at 1 h (D). The data are all displayed as the mean±s.d. and were analyzed by one-way analysis of variance. *P<0.05. Ra, average roughness; Rq, root mean square roughness.

In the topographic images obtained by AFM, the bright area was the elevated part of the cell, where the nucleus was (Fig. 4). The cell height, diameter and surface roughness were calculated by cross-sectional analysis of the height-measurement images. The cells in the control group extend well, and their surface was smooth. The texture of the actin bundles was clearly visible (Fig. 4B, 0 h). In contrast, the surface of treated cells became increasingly rough, the periphery of the cells became irregular and the area of cell extension gradually decreased. The texture of actin bundles disappeared (Fig. 4B). The height of the cell gradually increased (Fig. 4D). In addition, the control cells displayed the least roughness (Ra: 667±36 nm, Rq: 677±24 nm). The average roughness (Ra) and Root-Mean-Square roughness (Rq) values of the treated cells were significantly higher (*P*<0.05) than those of the control (Fig. 5A,B). These findings confirmed that the surface nanomorphology significantly changed after the cells were treated with CB, exhibiting an obvious time dependence. The height of the cells appeared to increase at first and then slightly decrease, but was always higher than that of the control (Fig. 5C). The cell volume continued to decline and was significantly decreased at 1 h (Fig. 5D). The results suggested that the morphology of cells at nanoscale significantly changed with the continuous depolymerization of F-actin and the collapse of the microfilament cytoskeleton.

### The cell elastic modulus declined

**Fig. 6.**
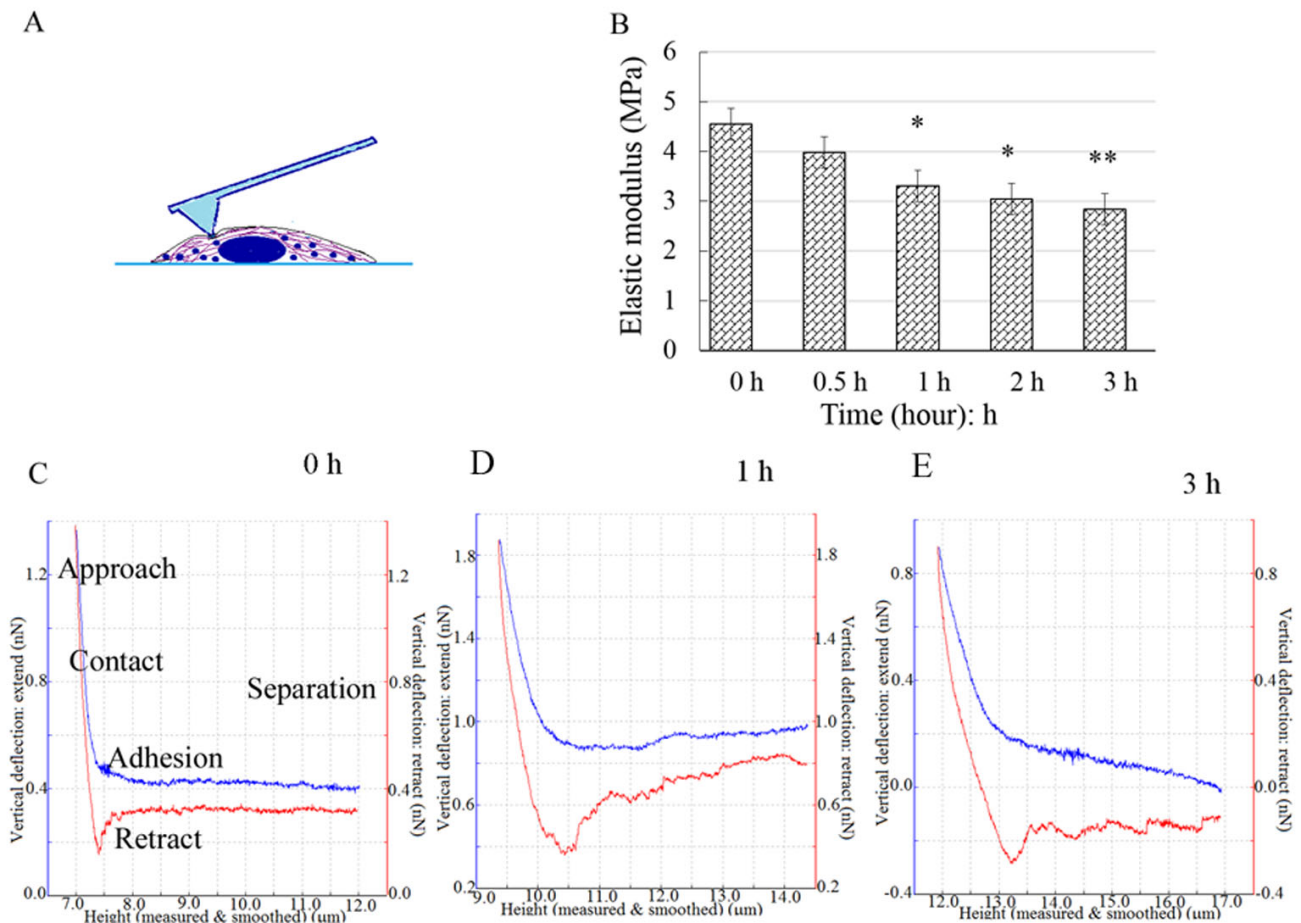
**The nanoindentation experiment, force spectra and elastic modulus analysis.** A schematic of the indentation experiment: the points were selected in a region rich in cytoplasm around the nucleus (A). The force curves were acquired by nanoindentation with AFM (C–E). The blue and red lines represent the approach curves and the retract curves, respectively. The elastic modulus *E* continuously declined and was significantly lower than that of the control at 1 h (B). The results are displayed as the mean±s.d. and were analyzed by one-way analysis of variance. *P<0.05, **P<0.01.

After the height-measurement image was obtained, 10–15 dots/cell were chosen to perform the nanoindentation experiments on (Fig. 6A), and the force-distance curves were acquired (Fig. 6C–E). The blue and red lines denote the approach curves and retract curves, respectively. The elastic modulus (*E*) of cells were quantified by analyzing the approach curves with Hertz model. Most of the *E*-values were distributed around the average value of each group. The average *E*-value was 4.55±0.88 MPa for the control cells. After the cells were exposed to CB, *E* continuously decreased to 2.84±0.33 MPa at 3 h (Fig. 6B), indicating that the cells increasingly softened during the apoptosis induced by CB.

The elastic modulus of a cell is mainly dependent on the microfilament framework of the cytoskeleton. The mechanical properties and nanomorphology of cells are regarded as indicators of cellular biological processes, such as malignant phenotypes, differentiation and mitosis. Our findings indicated that the elastic modulus and volume significantly decreased and the cell surface roughness obviously increased in initial 3 h of the CB treatment. In comparison, PS, which is a biologically early apoptosis marker, was still undetectable at that time. Therefore, the alterations in cell mechanical properties and nanomorphology occurred more rapidly than the PS exposure, implying that the reconstruction in mechanics and nanomorphology occurred in early apoptosis.

### The surface ultrastructure changed

**Fig. 7.**
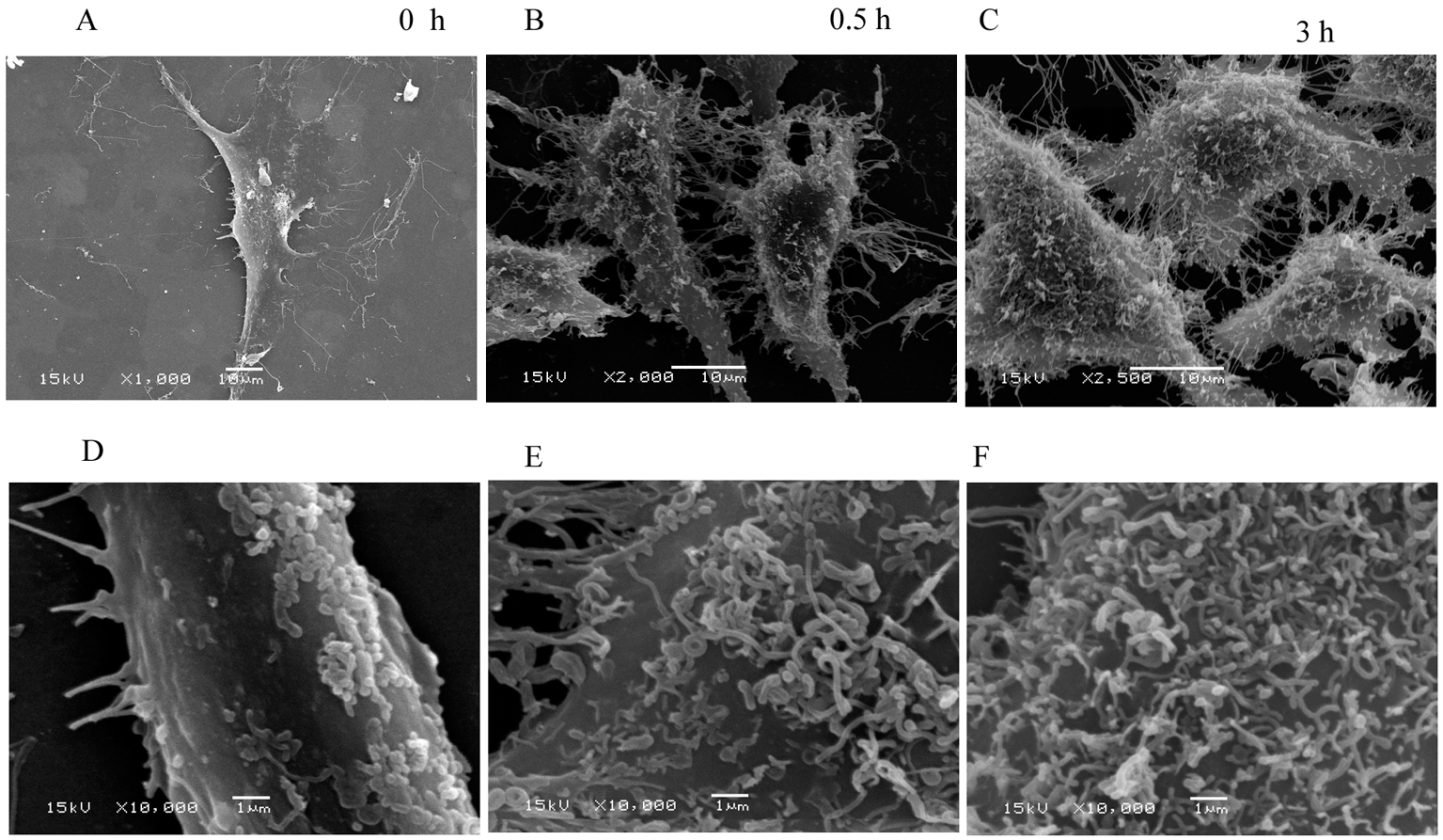
**The ultrastructural changes observed by scanning electron microscopy (SEM).** Overall views of the BMSCs at low magnification (A–C) and partial views at high magnification (D–F). The lamellipodia and filopodia were clearly visible around the control cells and a small number of particles were observed on the cell surface (A,D). In contrast, increasingly filamentous structures passed through the membrane and stayed on the cell surface during the cytochalasin B treatment (B,C,E,F).

The cell surfaces were smooth for the control group, and only a small number of dots were observed. The pericellular pseudopodia were clearly visible (Fig. 7A,D). In contrast, the surface of the cells in the treated groups became noticeably rough. A number of curved filaments passed through the membrane and stayed on the cell surface (Fig. 7B,C,E,F). These filaments may be responsible for the increased roughness of cell surface during the BMSC apoptosis. The filaments that passed through the membrane could be observed; however, whether these filaments were the broken F-actin fragments disrupted by CB could not be determined.

## DISCUSSION

In the present study, an apoptosis model of BMSCs induced by CB was established. The mechanical events and morphological changes at nanoscale were systematically investigated by AFM and SEM. In early apoptosis, the elastic modulus and volume of the BMSCs increasingly decreased while the roughness of the cell surface gradually increased, and numerous filamentous structures crossed the membrane and remained on the surface. These findings are consistent with the results of the stiffness, volume and surface roughness during the apoptosis of cancer cells induced by different factors in the literature (Zhang et al., 2016; Antico et al., 2013). However, these studies did not delve into why the surface of the cells became rougher. Our recent study investigated and carefully analyzed the causes of the increased surface roughness of cancer cells for the first time (Su et al., 2019).

The mechanical properties of a cell have been regarded as unique indicators that can directly reflect changes in the vitality state. In particular, alterations in elastic modulus or stiffness have been used as biological markers for cellular phenotypic events and diseases (Haghparast et al., 2013). In our study, the decrease in elastic modulus and volume suggested that although no obvious biological apoptosis was observed, the cell mechanical vitality was significantly altered within the first 3 h of treatment with CB. Other studies have found that microcantilever fluctuations can quantify cell viability and its changes in real time (Kim et al., 2011; Ndieyira et al., 2014). Therefore, the decline in elastic modulus and volume detected by the AFM microcantilever can reveal a decrease in cell viability in our study. Nikolaev and his colleagues investigated the elastic modulus among the viable, impaired membrane and dead cells labeled with Annexin V/PI and found that there were significant differences in the elastic modulus of these cells (Nikolaev et al., 2014). In contrast, our findings revealed that CB-treated viable cells that cannot be labeled by Annexin V/PI had undergone significant alterations in mechanics, nanomorphology and geometry. These results suggested that the vitality of these viable cells had changed, and the continuous decrease in cell vitality eventually led to apoptosis. In short, the decrease in elastic modulus and cell volume was consistent with exposure of the early apoptotic signal PS. Moreover, the alterations in mechanics, nanomorphology and geometry contributed to further understanding of the mechanical events involved in apoptosis and to monitoring the subtle changes in cell viability before the detection of biological apoptosis signals.

In addition, the cell surface roughness significantly increased with the F-actin cytoskeleton disrupted by CB. It is well known that cytoplasmic membrane blebbing can change the cell surface roughness during apoptosis. However, the membrane blebbing is mediated by caspase-triggered activation of the Rho effector protein ROCK I (Coleman et al., 2001). Caspases identified as the initiators and effectors or executioners of apoptosis were the products of middle and late apoptosis. Therefore, membrane blebbing must be a phenomenon of late apoptosis, rather than the cause of increased surface roughness in early apoptosis. Vijayarathna et al. reported that membrane blebbing was observed by SEM at about 12 h after apoptosis induction (Vijayarathna et al., 2017). This finding is consistent with the results that the caspases were obviously activated at approximately 16 h after the HeLa cells were treated with CB (Kulms et al., 2002; Hwang et al., 2013). However, in the present study the cell surface roughness significantly increased within 3 h of CB treatment. The ultrastructural morphology observed (Fig. 7) was also different from that of the membrane blebbing observed by Vijayarathna et al. (2017). In fact, fluorescence staining and its intensity analysis indicated that the F-actin cytoskeleton disrupted rapidly, and the F-actin fragments filled the cells after the BMSCs were treated by CB (Fig. 3). It is well established that eukaryotic cells are remarkably crowded with various biological macromolecules (Ellis, 2001). The F-actin fragments aggravated the degree of molecular crowding in the BMSC. According to Monte Carlo simulation, in a semipermeable biofilm vesicle, larger molecules tend to be redistributed to the peripheral region near the boundary of the vesicle, and some of them protrude across the wall due to the entropic compressing force, as the molecules become more crowded (Shew et al., 2014). Therefore, the increase in cell surface roughness might arise from the larger molecules protruding across the membrane in extremely crowded conditions.

In summary, the above data suggested that the cell mechanical alterations and morphological changes at nanoscale were mechanical events that occurred in early apoptosis of BMSCs. In the process of apoptosis, the cell elastic modulus and volume significantly decreased and the cell surface roughness obviously increased. Subsequently, the early apoptosis marker PS was detected and eventually the cells died. In combination with the previously described molecular mechanism of stem cell apoptosis (Tower, 2015; Liu et al., 2018; Sun et al., 2018; Chen et al., 2015; Cui et al., 2007), stem cell apoptosis is not only a biological apoptotic signal cascade process, but is accompanied by the reconstruction of mechanics and nanomorphology. These findings were helpful for further understanding and mastering the apoptosis of cells from the perspective of mechanics.

## MATERIALS AND METHODS

### Ethics approval

The Bioethical Committee of the Northwest Minzu University approved the use of the samples for this research.

### Reagents and cell culture

All reagents, including CB and fetal bovine serum (FBS), were obtained from Merck KGaA (Darmstadt, Germany) unless otherwise specified. An Annexin-FITC/PI apoptosis detection kit was acquired from MultiSciences (Hangzhou, China). Dulbecco's modified Eagle's medium/F-12 (DMEM/F-12) was purchased from HyClone (Utah, USA). Lymphocyte separation medium (LSM) and bovine serum albumin were obtained from Solarbio Science & Technology Company.

### Culture and identification of BMSCs

Approximately 3–5 ml bone marrow was extracted from a 3-month-old male goat anesthetized by laughing gas under aseptic conditions and was anticoagulated with 0.2 ml heparin. The marrow was washed with phosphate-buffered saline (PBS, pH 7.2) and centrifuged at 1500 r/min for 10 min. The supernatant was discarded and the cell pellets were resuspended in DMEM/F-12 medium supplemented with streptomycin, penicillin and 10% FBS. Then, the suspension was slowly added into the same volume of LSM. After centrifugation at 2200 r/min for 20 min, the bone marrow mononuclear cells were separated and transferred to a new sterile centrifuge tube to be washed three times. The cell pellet was resuspended in DMEM/F-12 medium supplemented with 100 μg/ml streptomycin, 100 U/ml penicillin, 10% FBS, 2 mM/l glutamine, 1% ascorbic acid and 1% nonessential amino acid solution and then plated in 25-cm^2^ culture flasks and cultured in a 37°C incubator containing 5% CO_2_. The medium was changed every 2 days. After approximately 7 days of culture, the cells reached 80–90% conﬂuence. The cells were detached by incubation with 0.25% trypsin–EDTA (Invitrogen, USA) and plated at a density of 1×10^4^ cells/cm^2^ for further culture. Cells at passage 3–5 were used to identify BMSCs by flow cytometric (FCM, BD FACS Aria, USA) detection of markers, including CD34, CD44 and CD45. BMSCs were detached with 0.25% trypsin-EDTA and collected by centrifugation. The pellets were resuspended with sterile PBS and adjusted to 1×10^6^ cells/ml. Approximately 1 ml cell suspension was absorbed and transferred to the EP tube. The cells were collected by centrifugation and resuspended with 200 μl PBS. Approximately 1 μl of monoclonal antibodies (CD45 PerCP-Cy5.5, ab210342; CD44-FITC, ab95138; CD34-Alexa Fluor^®^ 488, ab195013; 1:200 dilution, Abcam) were added to the cell suspension, and the same volume of PBS was added as a control. The specimens were incubated at 4°C for 30 min in the dark and were analyzed by flow cytometry. Three samples were prepared for each surface molecule of the BMSCs to be tested. BMSCs at passages 3–5 were used for the experiment.

### Biological viability and proliferation analysis

The Cell Counting Kit-8 (CCK-8) assay was used to estimate the biological cytotoxicity of the cells according to the manufacturer's instructions. The BMSCs were plated at 5×10^3^ cells/well in a 96-well plate and incubated overnight. Each group had three replicates (wells). The medium was substituted with fresh medium containing CB at various concentrations (0, 5, 10, 15 and 25 μg/ml) and incubated for 24 h. Then, 0.01 ml assay reagent was added to each well. After 1.5 h, the 96-well plate was analyzed using an enzyme-linked immunosorbent assay. The optical density of living cells was read at 450 nm in a microplate reader (Synergy HT; BioTek Instruments, Winooski, VT, USA).

### Apoptotic analysis of BMSCs by Annexin V-FITC/PI

During early apoptosis, the intracellular Ca^2+^ concentration increases, which causes the PS to translocate from the inner to the outer leaflet of the cell membrane (Suzuki et al., 2010; Martin et al., 1995). Therefore, the study applied the PS exposure to determine the early apoptosis and design the subsequent experiments. Annexin has a high affinity to PS and the translocation of PS was identified by an Annexin V. The early apoptosis was analyzed by FCM through the Annexin V-FITC/PI apoptosis detection kit. Approximately 5×10^5^ cells seeded on a 60-mm diameter Petri dish, incubated for 24 h, treated with CB for 0, 3, 6, 12 h and collected by trypsinization. The cell pellets were resuspended in 100 μl binding buffer and stained with 5 μl Annexin V-FITC and 5 μl PI for 30 min at room temperature (approximately 25°C) in the dark. The samples were analyzed with a FACSCalibur flow cytometer (BD Biosciences, Franklin Lakes, NJ, USA).

### F-actin cytoskeleton fluorescence staining and visualization

Cytochalasin B is a cytotoxic agent that interferes with the polymerization of actin filaments (F-actin). To visualize the alteration in F-actin framework, the BMSCs were grown on round coverslips (preplaced sterile coverslips in a 24-well cell culture plate) at a density of 4000 cells/cm^2^, stained with fluorescein isothiocyanate (FITC)-phalloidin. The specimens were rinsed with PBS, fixed with 4% cold paraformaldehyde, permeated with 0.2% Triton-X100 in PBS, blocked with 2% bovine serum albumin, and incubated with phalloidin for 1 h at room temperature in the dark. The nuclei were labeled with 0.1 mg/ml DAPI. The coverslips were sealed on glass slides with glycerin sealant, observed and photographed with a laser scanning confocal microscope (LSCM, Olympus FV1000, Japan) within a week. The mean fluorescence intensity was analyzed using FV10-ASW 4.1 Viewer software (Olympus, Japan). No filtering or adjustments were performed.

### Single-cell imaging and nanoindentation experiments

A bio-type Nano-Wizard III AFM (JPK Instruments, Germany) was employed to detect the topographical changes and quantify the alterations in the mechanical properties of living cells. The imaging and nanoindentation experiments were performed according to previously described operating procedures (Zhang et al., 2016; Su et al., 2019). The microscope was combined with an inverted optical microscope (Carl Zeiss, Germany) that was used to select the ideal cell. Silicon nitride cantilevers (PNP-DB, Nano World, Neuchatel, Switzerland) with a spring constant of 0.030 N/m (fo: 17 kHz) were applied. Prior to the measurement, the spring constants of the cantilever were calibrated with JPK Instruments software 4.2.61. A tip with a spring constant of 0.028–0.030 N/m was used in the subsequent experiments. The AFM imaging and nanoindentation experiments were implemented under intermittent mode using a square pyramidal tip with a 25° half-opening angle. When the tip intermittently interacts with the cells, the force-sensitive microcantilever fluctuated in three dimensions. The deflection was captured through the alteration of a laser projected on the cantilever. Approximately 1×10^4^ cells/dish were seeded in 35-mm Petri dishes. The experiments were conducted in cell culture medium at 37°C using a liquid temperature-controlled chamber. About 10–15 cells were imaged for each group. After topography scanning, 10–15 dots around the nucleus (this area is rich in cytoplasm, avoiding the influence of the substrate) were chosen to acquire force-distance curves by indentation. The interactions between the tip and the sample caused the cantilever to deflect, which was recorded as a function of the relative sample position, that is, a force-distance curve. The elastic modulus *E* (cell stiffness) was calculated through analysis of a series of 130–150 curves with JPK Instruments data processing software.

### Quantitative analysis of alterations in nanomorphology and geometry

The height of the cell was defined as the distance between the top and bottom of the cell. The height, diameter and surface roughness were quantified by cross-sectional analysis of the height-measurement images. The Ra and Rq are crucial parameters for understanding the surface morphology of living cells at the nanoscale. The roughness provided quantitative data regarding the reconstruction of surface nanomorphology after the F-actin cytoskeleton was disturbed. The cellular volume is also another important indicator for the state of cell viability. The cell was regarded as half an oblate ellipsoid, its volume can be calculated according to the following equation (Hessler et al., 2005):(1)
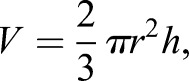
where *V* is the volume, *r* is the radius and *h* is the height.

### Elastic modulus measurement

The height-measurement images and the force-distance curves were obtained by the sensitive cantilever fluctuations. The cell was considered an elastomer of homogeneous structure. Thus, the cell elastic modulus *E* was calculated according to the Hertz model and the approach curves (Zuk et al., 2011; Geiger et al., 2009). The referential equation that gives the relation among indentation force, elastic modulus and depth is as follows:(2)
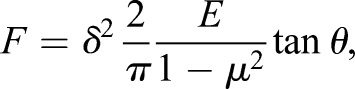
where *E* is the elastic modulus, *F* is the loading force, *μ* is the Poisson's ratio of the samples, *δ* is the indentation depth and *θ* is the half-opening angle of the tip.

### Cell surface observed by SEM

Prior to obvious biological apoptosis, the surface roughness measured by AFM significantly increased. To further elucidate the phenomenon, the cells were observed by SEM. Approximately 2–5×10^3^ cells were seeded onto sterile coverslips in a 24-well cell culture plate, cultured for 24 h, and treated with CB. The samples were rinsed with PBS, fixed with precooled glutaraldehyde solution at 4°C overnight, and made into ultra-thin slices. The cells were observed and captured by SEM (JSM6380 LV, Japan).

### Statistical analysis

The data were recorded as the mean±standard deviation (s.d.) and analyzed using SPSS 22.0 (Statistical Product and Service Solutions, Stanford University, CA, USA). Statistical differences were performed using one-way analysis of variance. *P-*values of <0.05 were considered statistically significant differences. A single asterisk (*) indicates significant difference (*P*<0.05), and double asterisks (**) denote extreme statistical difference (*P*<0.01).
